# Review: Alternative Splicing (AS) of Genes As An Approach for Generating Protein Complexity

**DOI:** 10.2174/1389202911314030004

**Published:** 2013-05

**Authors:** Bishakha Roy, Larisa M Haupt, Lyn R Griffiths

**Affiliations:** Genomics Research Centre, Griffith Health Institute, Griffith University Gold Coast, Queensland 4222, Australia

**Keywords:** Alternative splicing (AS), Bioinformatics, Database, Expressed sequence tags (ESTs), Microarray, mRNA, Protein, Splice variants.

## Abstract

Prior to the completion of the human genome project, the human genome was thought to have a greater number of genes as it seemed structurally and functionally more complex than other simpler organisms. This along with the belief of “one gene, one protein”, were demonstrated to be incorrect. The inequality in the ratio of gene to protein formation gave rise to the theory of alternative splicing (AS). AS is a mechanism by which one gene gives rise to multiple protein products. Numerous databases and online bioinformatic tools are available for the detection and analysis of AS. Bioinformatics provides an important approach to study mRNA and protein diversity by various tools such as expressed sequence tag (EST) sequences obtained from completely processed mRNA. Microarrays and deep sequencing approaches also aid in the detection of splicing events. Initially it was postulated that AS occurred only in about 5% of all genes but was later found to be more abundant. Using bioinformatic approaches, the level of AS in human genes was found to be fairly high with 35-59% of genes having at least one AS form. Our ability to determine and predict AS is important as disorders in splicing patterns may lead to abnormal splice variants resulting in genetic diseases. In addition, the diversity of proteins produced by AS poses a challenge for successful drug discovery and therefore a greater understanding of AS would be beneficial.

## 1 INTRODUCTION

With the completion of the human genome project, new insights into genomic data have been made possible. Humans produce around 90,000 different types of proteins, hence it was assumed that a corresponding number of genes would be present in the human genome [1]. It was also reasoned that since the human genome is structurally and functionally more complex than other simpler organisms, this would correspond to a proportionally higher number of genes. However it came as a surprise to discover that humans had fewer than 25,000 genes while a much simpler organism such as corn (*Zea mays)* contains approximately 40,000 genes [1]. This acknowledged mismatch in the ratio of gene to protein formation and the accompanying ‘one gene’ to ‘one protein’ paradigm resulted in the concept of alternative splicing (AS) of genes.

In order to explain the small number of human genes discovered, several hypotheses have been postulated. One suggestion was that since the human genome is more complex than other multicellular organisms, a greater level of regulation of genes and pathways would be involved [2, 3]. Another hypothesis suggests that post-translational modifications, which consist of more than 200 different types, could produce a number of different protein products. A third hypothesis relates to alternative splicing. AS is another mechanism by which a single gene could give rise to multiple protein products adding to protein diversity [3, 4].

Although the concept of AS was first introduced in the 1970s, only a few hundred AS genes have been recognized to date. In 1977, introns and exons were discovered in the adenovirus *hexon *gene. It was predicted by Walter Gilbert that different arrangements of exons could be spliced together (AS) to give diverse mRNA isoforms [5]. It was assumed that around 5% of genes in higher eukaryotes undergo AS [6]. High-throughput sequencing of the human genome, especially via analysis of expressed sequence tag (EST) sequences has provided a different approach for the analysis of AS through bioinformatics. As ESTs are obtained from completely processed mRNA, they present extensive mRNA diversity. From the year 2000 onwards, bioinformatic studies have progressively identified a magnitude of AS in genes than were previously known and are beginning to provide a global view of AS in humans [5]. More than 90% of pre-mRNA is detached as introns and only about 10% of the average pre-mRNA is connected as exonic sequence by the splicing of pre-mRNA. Although AS was initially assumed to occur in about 5% of all genes [6], it was soon realized that this mechanism was vastly more abundant [7]. AS occurs in all tissues, but the most common occurrence of tissue-specific splicing is observed in brain cells [8, 9]. Via EST data comparisons, the process of AS can be seen in evolutionarily discrete species such as humans, mice, *Drosophila* and *C. elegans*, signifying a plausible role for AS in evolution [3, 7].

Much research has focused on the genome-wide identification of AS events in various cell and tissue types, under different conditions to ascertain the degree of functionally significant AS events. Sequence-based and microarray-based analyses have shown that AS events are more commonly observed in transcripts present in those genes of functionally complex tissues having varied cell types like the brain and testis [10]. These AS events also commonly occur in genes in cell types that participate in the selection and supply of diverse functions, such as the immune system [11-13]. Several complex examples have been identified including the neurexin and CD44 loci, which have two or more AS sites, with the autonomy of AS cassettes thought to form several protein accumulations encoded by a single locus [14, 15]. Another example is the *Drosophila *gene Dscam, which encodes the axon guidance receptor and is able to produce approximately 38, 016 different protein isoforms [14, 16].

Importantly, when cells alter their AS pattern in response to a signal, protein variants with varied biological functions may be formed. When protein complexes are formed on processed pre-mRNA, it helps in the selection of AS sites. It has now been demonstrated that reversible phosphorylation shows a strong effect on pre-mRNA processing [17]. It is likely that AS variants that code for unstable protein domains are nonfunctional because the encoded protein is unlikely to fold to its expected unique structure. Reverse transcriptase-polymerase chain reaction (RT-PCR) experiments have demonstrated that splice variants coding for unstable proteins are less abundant than those that code for stable proteins [18]. From the abundance of AS seen in higher eukaryotes, it is probable that many features of the cell phenotype, including those that lead to tumour development, are monitored by the relative expression of the AS isoforms of several genes [19].

### 1.1 Mechanism of Alternative Splicing

The process of splicing occurs in the spliceosome, a complex of five small nuclear RNAs, associated core proteins and many hundreds of proteins that gather on nascent pre-mRNAs during transcription. The splicing mechanism is an organized series of congregation and conformational reorganization events, interrupted by the chemical transformations of the cleavage of phosphodiester bonds at exon/intron junctions and the formation of a phosphodiester bond when the exon is ligated [20]. This model, referred to as the combinatorial model, suggests that AS in mammalian cells is mainly controlled by the binding of general splicing factors to pre-mRNA molecules when the spliceosome is produced. The spliceosome has most of these factors, including a class of proteins called serine/arginine-rich (SR) proteins, which have one or two RNA-binding domains and a protein-protein interaction domain, rich in serine and arginine amino acids. This model also suggests that AS transcripts employ various combinations of pre-mRNA splicing factors *in vivo*. The selection of splicing factors employed for each spliceosome depends on both the concentration of each factor in individual cell types and the regulatory elements present in each pre-mRNA [21].

The detection of splice sites and pre-mRNA splicing are dynamic processes that require steady remodelling of proteins and ribonuclear protein units on the pre-mRNA being processed. They are related to other processing links including transcription, 5’-end capping, 3’-end polyadenylation and nuclear export. When splicing regulatory proteins remain attached to their pre-mRNA, they frequently participate in several processing events [17, 22]. Splice sites bordering exons in higher eukaryotes are vastly degenerate and are inadequate for accurate exon recognition. Thus protein complexes are generated on the pre-mRNA and aid in the detection of splice sites with high precision. The majority of these splicing regulatory proteins are defined as two major classes namely heterogeneous nuclear ribonucleoproteins (hnRNPs) and SR proteins. These proteins include domains for RNA-binding and protein/protein interactions. They bind with low specificity to the available and generally single-stranded component of pre-mRNA [23]. For enhancing RNA binding specificity, splicing regulatory proteins utilize their protein interaction domains to bind to each other. Protein/protein interactions in these proteins also experience a low specificity. After the formation of these protein complexes around an exon, ribonuclear protein components of the core spliceosome generate RNA/RNA interactions at the 5’-splice site and at the branch point [17]. These mechanisms have aided our understanding of the factors affecting splicing regulation.

### 1.2 Types of AS

**Fig. (1):**
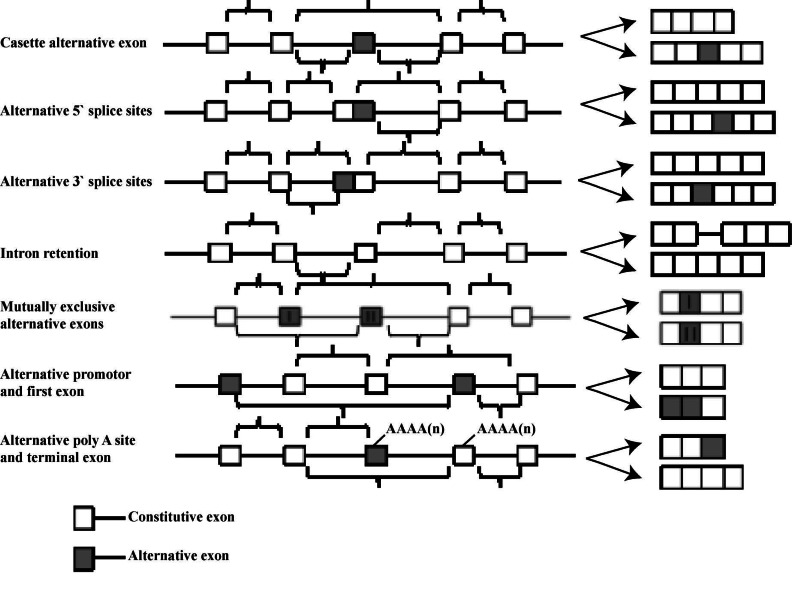
Types of AS commonly observed adapted from Blencowe [10].

Currently, there are seven main types of AS described. Of these, the cassette-type alternative exon account for approximately one-third of all AS events. The alternative 5’ or 3’ splice sites commonly occur together, forming approximately 25% of all AS events and are proficient enough to bring about changes in the coding sequence in as little as one codon. All AS events summarised may occur in the translated as well as the untranslated regions of the transcripts [24] (Fig. **1**).

## 2 CLINICAL RELEVANCE

Abnormal splice variants can be formed due to disorders in the splicing patterns and this is observed in more than 50% of genetic diseases including cancer. Tumorous growth is favoured by distortion of the splice sites in a neoplastic cell. Due to mutations or polymorphisms in the gene, RNA splicing can be dysregulated and this becomes part of the acquired pathology. Around 15% of point mutations linked with genetic disorders are credited to aberrant splicing [25], with several diseases linked to mutations that contribute to abnormal regulation of AS resulting in the expression of irregular protein isoforms for the specific cell type [26, 27].

It has become evident in recent years that successful drug discovery needs an understanding of the complexities associated with the associated biological background. In terms of proteomics, AS generates an added layer of complexity. In recent years, there has been great progress in obtaining information about AS. Despite these advancements, core drug discovery processes involve techniques unable to differentiate between splice variants and are thus trapped in the ‘one gene - one protein’ theory. As such, when AS is not taken into consideration, drug discovery is limited to a small proportion of the targets discoverable via proteomics and thus may miss many possible protein targets. Even when studies are carried out focusing on a specific gene, results will be prejudiced if AS is ignored [28]. Alteration in splicing account for many diseases including β-thalassemia (an autosomal recessive disorder) related to mutations in the second intron of the β-globin gene [29]. Mutations near splice sites may alter the transcript splicing pattern either by abnormal inclusion or exclusion of exon(s) and/or variations of 5’ or 3’ sites [30]. Variation in the 5’ splice site activates a common 3’ cryptic site upstream of the mutations. It also stimulates inclusion of a fragment of the intron-containing stop codon resulting in a decreased amount of functional β-globin protein [29].

Cancer is often a result of errors in pre-mRNA splicing leading to altered gene expression patterns. Affected proteins can include transcription factors, cell signal transducers and components of the extracellular matrix. For diagnostic purposes, changes in splicing patterns can be used as markers of the cell linked with disease. Some therapeutic tactics making use of AS include the use of protein over-expression to alter splicing of the affected exon [31], using antisense oligonucleotides [32], siRNA-based drugs for silencing gene expression [33], using compounds affecting phosphorylation of splicing factors [34], high-throughput screening to recognize compounds affecting splicing efficiencies of target pre-mRNAs [35] and restoring mutated exons with wild-type exons [36].

Another example of a disease caused by alterations in the splicing patterns is the microtubule-associated protein tau (encoded by the *MAPT *gene). This protein undergoes AS to produce six protein isoforms in the human brain [37, 38]. Mutations that cause abnormality in the splicing elements in the *MAPT* gene produce irregular ratios of *MAPT* transcripts observed in patients affected with an inherited form of dementia. In 1994, an inherited form of fronto-temporal dementia with Parkinsonism was related to chromosome 17 (FTDP-17) [39]. It was later found that mutations in *MAPT* produced abnormal tau protein causing neurodegeneration, demonstrating a role for *MAPT* in FTDP-17. There are two types of mutations in tau causing FTDP-17 of which the first is either a missense or deletion mutation in the tau protein and the second, affects AS of exon 10 in tau, causing an increase or decrease in exon 10 ultimately leading to FTDP-17 [40-42]. In addition, exons 2 and 3 along with exon 10 in the tau pre-mRNA, undergo AS to generate isoforms that differ in their N- and C- terminals. Exons 2 and 3 undergo AS in the N- terminal half, generating three separate mRNA molecules, which skip both exon 2 and 3, include only exon 2 or include both exons 2 and 3. In the C-terminal half, exons 9 to 12 encode four microtubule-binding domains which are repeats of 31 or 32 amino acids [43]. In the N-terminal half, exon 10 may be included or skipped independently of AS, generating three-microtubule repeats (3R, exon 10^-^) or the four-microtubule repeat (4R, exon 10^+^) isoforms of the protein. Under normal circumstances, the ratio of 4R to 3R is approximately equal to one and this balance is essential for proper neuronal function [41, 42, 44].

To further understand the significance of precise AS patterns required for the normal functioning of a cell, *MAPT *pre-mRNA serves as a good model as when alternatively spliced, six isoforms of the tau protein are produced [45]. Specifically, AS in exon 10 of this gene is regulated through the complex interaction of exonic splicing enhancers (ESEs), exonic splicing silencers (ESSs), intronic splicing enhancers (ISEs) and intronic splicing silencers (ISS) [46, 47]. The mutation (N279K) in exon 10 causes transversion from T →G (U→G in the transcript). N279K increases exon 10 inclusion by fortifying a purine-rich ESE, which enhances the sequence from AA**U**AAGAAG to AA**G**AAGAAG [46, 48]. Another mutation, Del280K further assists the function of the enhancer. This mutation causes a deletion of an AAG repeat from the ESE, leading to decreased exon 10 inclusion and also a decreased 4R/3R tau ratio [46]. The ESE engages Tra2β (transformer 2 beta homolog - Drosophila) to bind the AAG repeat and augments the inclusion in exon 10 [48]. A silent mutation (L284L) occurs downstream of the ESE and causes a disorder of the ESS (U**U**AG to U**C**AG), enhancing exon 10 inclusion [46]. Intron 10 is also a host to many other mutations occurring immediately downstream of the 5' splice site. These mutations may cause destabilization of a stem-loop structure that forms between the end of exon 10 and the 5' splice site. This destabilization is thought to lead to enhanced recognition of exon 10 by the spliceosomal U1 snRNP (U1 small nuclear ribonucleoprotein), leading to increased exon 10 inclusion [40-42, 46]. The significance of altered regulation of exon 10 inclusion in FTDP-17 cases is emphasized by both the number and the range of potential mutations [45].

Another example of the significance of AS in accurate cellular functioning is the cellular processes associated with cell survival through the production of the promoter of apoptosis Bcl-xS instead of a suppressor, the splice variant Bcl-xL. In the daily activities of cells, AS is ubiquitous, influencing and affecting central processes including intracellular signaling and structural phenotype [1]. It is also important to note that there is a high level of conservation in intron sequences that border conserved AS events. This highlights the need for regular inspection of these regions in diseased genes for mutations that alter splicing patterns.

### Insight into Cancer Therapeutics

The occurrence of splicing events varies dependent upon cell type, developmental stage and disease state, including cancer. Metastasis in cancer cells is an active process and may occur as a result of genome variability and flexible proteomes caused by AS affecting the cell growth and survival mechanisms. In addition, cancer cells may themselves produce AS events in order to sustain their population and/or metastasise. As such, it is conceivable that AS events may play an important role at all stages of tumourigenesis. Our ability to identify and differentiate between AS variants as biomarkers in normal and cancerous cells will aid in better understanding and treating all cancers [49].

In 2008, Thorsen *et al.* [50] performed whole genome exon expression of 102 normal and cancer tissue samples of different stages from colon, urinary bladder and prostate cancers using the GeneChip Human Exon 1.0 ST Array to examine tissue and tumour-specific AS. In the study, 2069 candidate AS events were detected between normal tissue samples from colon, bladder and prostate. From these, 115 splicing events were selected for validation using RT-PCR and sequencing, with 10 events successfully validated. They further examined several candidate tumour-specific variations from colon, bladder and prostate cancer for validation using RT-PCR in an independent set (n= 81) of normal and tumour tissue samples. Using this replication population of samples, they were able to detect seven genes with tumour-specific splice variants (ACTN1, CALD1, COL6A3, LRRFIP2, PIK4CB, TPM1 and VCL). The validated tumour-specific variations were found to be consistent to clearly distinguish between normal and cancer samples and in some cases different tumour stages. A subgroup of the tumour-specific splicing variations (ACTN1, CALD1, and VCL) was observed in all three organs and may indicate general cancer-related splicing events [50]. In other studies, antisense oligonucleotides have been applied to down-regulate mRNAs resulting in improved cancer cell survival and to modify splicing patterns in muscular dystrophy, revealing promising results in the clinical investigations [51].

Most chemotherapeutic interventions against cancer utilize the induction of an apoptotic response but to date, there is minimal information available about the effect of chemotherapeutic agents on the AS of the apoptotic regulatory genes. A study in 2008 by Shkreta *et al.* [52] tested 20 mainstream anticancer drugs for their ability to mediate the production of Bcl-x splice isoforms. Using 293 cells, several drugs shifted splicing toward the proapoptotic Bcl-x_S_ splice variant. Several drugs also shifted Bcl-x in the cancer cell lines (MCF-7, HeLa, PC-3, PA-1, and SKOV-3) however the set of active drugs differed between cell lines. They also observed that almost every drug could modify a subset of AS events in each cell line and indicated cell line-specific differences may occur in the pathways that control AS [52]. A major problem in cancer treatment is resistance to chemotherapy. Tumours are frequently sensitive to chemotherapy upon early treatment but can develop resistance over repeated treatments, with some cells able to survive and promote cellular mechanisms boosting their resistance to subsequent chemotherapy. Acknowledging the role of AS in regulating the apoptotic response to chemotherapy, research is now examining novel ways of regulating cellular control of key AS genes. There have been some advances in the development of chemotherapeutics that directly target AS in specific genes to regulate the protein isoform that will be formed including splice switching oligonucleotides which are designed to attach to pre-mRNA and prevent splice site utilization at the binding site [53].

## 3 DETECTION OF AS

### 3.1 Using Expressed Sequence Tags (ESTs)

EST and cDNA sequences can be aligned to genomic sequences using programs that explore conserved splice-site consensus sequences flanking the gaps formed by intron sequences between the aligned exons [10]. Huge databases of AS events extracted this way have been created for several species including humans, mice and rats [5, 54, 55]. However, there is an essential limitation to AS analyses using transcript sequence data. EST coverage is classically biased towards the 3′ and 5′ ends of transcripts with a general inadequacy in the number of sequenced transcripts available to determine the frequency of either the incorporation or exclusion of alternative exons in a given cell/tissue, or under specific experimental conditions [13]. The effect of AS-induced transcript variations may cause a complete loss of protein function along with other effects such as variation in transcript localization, stability and translation [56-58]. This suggests that AS not only diversifies protein function, but also gives a level of control during the development and function of healthy tissue [59].

### 3.2 Using Microarrays

**Fig. (2):**
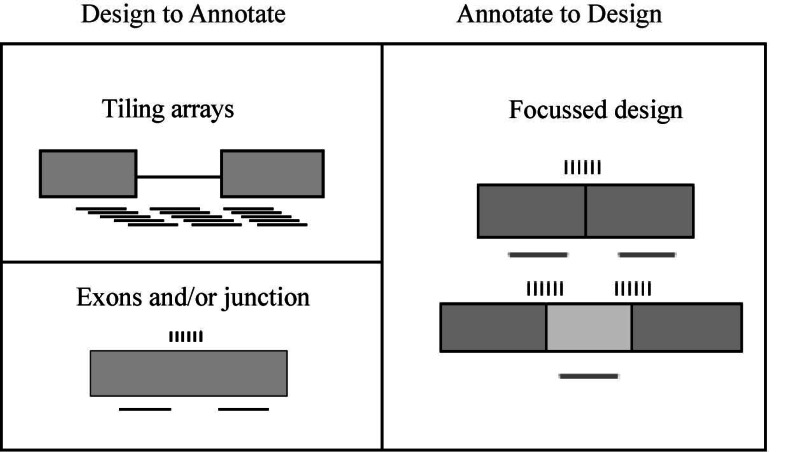
Methods used to design AS microarray probes adapted from Cuperlovic-Culf *et al*. [63].

Microarray data helps in predicting splicing events with these predictions able to be applied to direct RT-PCR at specific transcript locations. These are useful for the observation of tissue-specific gene expression at the genomic level along with the detection and quantification of isoforms [19]. It has also been demonstrated that microarrays can be used to examine pre m-RNA splicing [57, 58]. There are two main classes of microarrays that are used in probe design for detecting and quantifying diverse AS forms. The first includes ‘annotate-to-design’ methods [60, 61], the second the ‘design-to-annotate’ methods [62]. These can be further categorized into three subclasses of microarray platforms namely ‘tiling’, ‘exon/junction’ and ‘focused’, to detect and quantify mRNA splice variants (Fig. **2**).

#### Tiling Arrays

Tiling arrays offer a complete coverage of the genome and are thus useful in the discovery of new splice variants while the disadvantage of this platform is that it requires a large number of probes.

#### Exons and/or Junction

The exons and/or junction platform offers some flexibility in the position of probes allowing the probe characteristics to be more homogenous. The disadvantages of this system include that it is mainly restricted to type I deletions (cassette exons) and known information of exon boundaries is a prerequisite. Junction arrays are useful as they support tissue-specific AS in thousands of genes and allow the identification and verification of splice variants not currently represented by ESTs or mRNAs. However, there are some limitations to using junction arrays. Similar to ESTs, junction arrays cannot establish if two splicing events in one tissue are in the same or different transcripts, and novel isoform sequences are not described. Cross-hybridization may also generate false positives when genes with similar sequences have strong tissue-specific regulation [19].

#### Focussed Design

The focussed design platform utilises a more simple data analysis requiring a smaller number of probes and also allows the quantitative determination of known splice forms. Disadvantages of this platform include the limitation of the design to known splice forms and the inclusion of errors due to unknown splice forms. The common disadvantages of these three platforms are that the analysis is difficult, there may be variations in probe characteristics and the occurrence of cross hybridization [19].

### 3.3 Other Microarray Platforms

#### Splice-Variant Oligonucleotide Microarray

Splice-variant oligonucleotide microarray is a high-throughput platform used to monitor splice-variant expression for therapeutics and diagnostics. Isoform-specific differential expression data can be obtained through the observed difference in hybridization signal between two oligonucleotides within the same gene. Genomic tiling arrays and exon arrays are used to recognize co-regulated exons allowing the inference of variant mixtures [64]. Expression arrays with many probes are examined to recognize exons differentially added or excluded in a tissue-specific manner. The RNA-mediated ligation pooled with array technologies, gives a novel approach for identifying exon-exon junction information of known splice variants.

#### Spotted Oligonucleotide Microarrays

Spotted oligonucleotide microarrays using probes designed to identify unprocessed and processed RNA have been utilized to screen pre-mRNA splicing in yeast [65] and the processing of non-coding RNAs in yeast and mammalian cells [66].

### 3.4 Other Tools

Other methods including a fiber-optic based arrays [67], polymerase colony assay [68] and other microarray-based methods using spotted cDNA fragments or oligonucleotides [40] have also proved useful for observing AS in mammalian cells. In order to detect AS in various tissues, it is imperative that experimental tools capable of identifying AS patterns directly and on a large scale are readily available [24]. A limitation of the microarray technique used for detecting AS is that long cDNAs cannot identify mRNA isoforms that differ by a micro exon or by the substitution of an exon of similar sequence [69].

#### RNA-Mediated Annealing, Selection and Ligation (RASL)

RNA-mediated annealing, selection and ligation (RASL) is an excellent tool to detect these minor sequence differences. RASL requires the annealing of total cellular RNA samples with a mixture of oligonucleotides complementary to sequences on both sides of the splice junction. This is followed by poly (A)^+^ selection, ligation of flanking oligonucleotides and then amplification of the ligated oligonucleotides by PCR. PCR products are fluorescently labelled and examined on microarrays [24].

#### Surface Enhanced Raman Scattering (SERS)

Surface enhanced Raman scattering (SERS) based detection using nonfluorescent labels can be used to examine AS events. This method is a sensitive and selective tool for complete AS profiling of genes that are relevant to specific diseases [70].

#### Bead-Based Fiber-Optic Microarray System

In 2002, the novel technology of a bead-based fiber-optic microarray system able to analyze both gene expression and AS analogously was developed [67]. Due to its specificity, the assay is able to distinguish mRNA isoforms within a single gene. Limitations of this method include selection of the most suitable sequence for hybridization, difficulty in screening for novel transcript expression and difficulty in analyzing mRNA isoforms when many regions in a particular transcript undergo AS. This approach is most suitable for large-scale analysis of genomic surveys in large databases. The cDNA arrays also have their limitations as their long probes cannot identify the minor sequence differences that may occur due to insertion of nucleotides or the substitution of related exons [67]. Out of the techniques described for detecting AS, the ideal method is selected based on experimental design.

#### Deep Sequencing Technology

Unlike microarray methods, sequencing-based methods directly determine the cDNA sequence. Initially, sequencing reactions were performed using Sanger sequencing of cDNA or EST libraries [71]. However this method is comparatively low throughput, expensive and usually not quantitative. Tag-based approaches were then developed such as serial analysis of gene expression (SAGE) [72] cap analysis of gene expression (CAGE) [73] and massively parallel signature sequencing (MPSS) [74]. These methods are high-throughput and can offer precise, 'digital' gene expression levels. Nevertheless, most are still based on expensive Sanger sequencing technology and a large portion of the short tags cannot be uniquely mapped to the reference genome. In addition, only a portion of the transcript is analyzed and isoforms are commonly difficult to distinguish from each other. More recently, the development of novel high-throughput DNA sequencing approaches have offered new means for mapping and quantifying transcriptomes. This method, termed RNA-Seq (RNA sequencing) provides a more accurate measurement of transcript levels and their isoforms. In this technology, a library of cDNA fragments with adaptors attached to one or both ends are obtained from a population of RNA (total or fractioned, such as poly (A)+). High-throughput sequencing is carried out to obtain short sequences from one end (single-end sequencing) or both ends (pair-end sequencing) from each molecule, with or without amplification [75]. Depending on the DNA sequencing technology used, including Illumina IG [76], Applied Biosystems SOLiD [77] and Roche 454 Life Science [78], the read lengths attained are generally in the 30-400bp range. After sequencing, the acquired reads are either aligned to a reference genome/reference transcripts or gathered de novo without the genomic sequence to generate a genome-scale transcription map that includes both the transcriptional structure and/or level of expression for each gene [75].

#### Advantages and Challenges of Deep Sequencing Technology

One of the main advantages of RNA-Seq over hybridisation-based methods is that it is not limited to identifying transcripts corresponding to existing genomic sequences. As an example, using the 454-based RNA sequencing, transcriptome sequencing of the Glanville fritillary butterfly has been attained [79]. RNA-Seq can also determine the exact location of transcription boundaries down to a single-base resolution. Using the 30-bp short reads obtained from RNA-Seq, information about the connectivity of two exons can be acquired and longer reads or pair-end short reads can reveal connectivity between multiple exons, making RNA-Seq a useful tool for analyzing complex transcriptomes. In addition, information about sequence variations (for example, SNPs) in the transcribed regions can also be obtained from RNA-Seq [80].

Another important advantage of RNA-Seq over DNA microarrays is that RNA-Seq has minimal background signal as DNA sequences can be clearly mapped to unique regions of the genome. This methodology has also been found to be accurate in quantifying expression levels by quantitative PCR (qPCR) [81], demonstrated by spike-in RNA controls [76]. RNA-Seq data also has a high level of reproducibility for technical as well as biological replicates [81]. In addition, with the absence of cloning steps as well as the absence of any amplification steps due to the Helicos technology, RNA-Seq also requires less starting RNA [75].

Although RNA-Seq involves only a few steps, there are several manipulation steps in the cDNA library preparation stage, which may complicate its use in profiling all types of transcripts. As a high-throughput sequencing method, RNA-seq faces the challenge to develop efficient retrieval and processing of large amounts of data while minimizing errors in image analysis, base-calling and low-quality reads. When processing large transcriptomes, a key problem is when a major portion of the sequence matches multiple locations within the genome. Short reads with high copy numbers (>100) and long portions of repetitive regions have proved to be challenging to this technology [75]. One solution to overcome the multi-matching problem is to acquire longer reads or instead of a paired-end sequence approach where short sequences are obtained from both ends of a DNA fragment [82], extending the mapped fragment length to 200-500bp. Additionally, sequencing errors and polymorphisms can provide mapping challenges for all genomes, not only for repetitive DNA. Usually, single base changes are not problematic as most mapping algorithms accommodate one or two base differences. Nevertheless, larger differences may cause problems and will need better reference genome annotation for polymorphisms and deeper sequence coverage. Greater sequence coverage screening entails more sequence depth, while in order to determine a rare transcript or variant, extensive depth is essential. Usually, the larger the genome, the more complex the transcriptome and the more sequence depth is needed for adequate coverage [75].

## 4 BIOINFORMATIC APPROACHES

A significant focus of research in bioinformatics has centered on the development of useful AS databases for the analysis of AS patterns in genes. The most common bioinformatics method employed is the use of ESTs to determine differences such as large insertions or deletions. This enables further analysis by placing the ESTs within the gene sequence. In this way, the matched sequence will form the candidate exon while candidate splices will have large gaps in the EST-genomic alignment. Around 145 genes were described by ‘The International Human Genome Sequencing Consortium’ to be alternatively spliced, following widespread analysis on chromosome 22 by aligning ESTs with the genomic sequence [5, 83]. The level of AS in human genes was found to be between 35 to 59% of genes having at least one AS form [83] with estimates that 70–88% of AS alter the protein product [83] such as substitution of the amino or carboxyl terminus, in-frame addition and removal of a functional unit. As only a few ESTs have been sequenced for most genes, it is likely that many more AS forms may exist and are yet to be detected [5].

### Bioinformatic Tools

Some of the existing databases and bioinformatics tools related to AS detection and analysis are described below. The online database ASAP (Alternative Splicing Annotation Project) is a valuable tool for biologists to obtain vast amounts of AS information from genomic and proteomic applications. ASAP commenced in 2002 and currently supplies AS data for human genes. A genome-wide analysis of several other organisms (Arabidopsis, mouse etc.) is currently being carried out. ASAP gives accurate gene exon-intron structure, AS analysis, tissue specificity of AS forms and the protein isoform sequences arising as a result of AS, providing protein isoform sequences for each splice form. ASAP employs EST alignment to the sequences for AS detection and exact sequence information for the resultant change in the sequence [55]. It also gives results from genome-wide analysis of tissue specific human AS forms [9].

The Alternative Splicing Gallery (ASG) represents the huge quantity of EST and cDNA data at the genomic level. It serves as an initiation point for the systematic examination of gene structure and the transcriptome. ASG has integrated transcript data from RefSeq, Ensembl, UniGene, STACK and TIGR with Ensembl genes clearly displayed into splicing graphs for human genes. However, ASG does have several limitations. The database may over predict transcript number when there are dependences between AS events in a gene (e.g. events which always occur together or never occur together). Also, it does not show annotations of coding sequences, promoters, polyadenylation sites, strength of the splice sites and transcript truncations [84]. Another AS database called ‘HOLLYWOOD’ was constructed using the genomic annotation of splicing patterns of known genes obtained from the spliced alignment of cDNAs and ESTs. It shows many options including splice site sequences, exonic splicing enhancers and silencers, conserved and non-conserved patterns of splicing and complementary DNA (cDNA) library data for the queried alternative exons. It currently holds comprehensive information for human and mouse genes [85]. The alternative splicing database (ASD) consortium is methodically gathering and annotating information on AS and consists of three databases namely AltSplice, AltExtron and AEdb [86]. The largest part of ASD is AltSplice, a database of computationally defined AS events. This database has information regarding AS introns/ exons, events, isoform splicing patterns and isoform peptide sequences. Data is gathered by investigating gene-transcript alignments. AEdb is the manually produced element of ASD. It contains sequence and properties of AS exons, a functional account of observed splicing events, characterization of observed splicing regulatory elements and a compilation of experimentally elucidated minigene constructs. ASD data has been included with the Ensembl genome annotation project as a ‘Distributed Annotation System’ (DAS) resource and can be accessed via the Ensembl genome browser [86].

The Manually Annotated Alternatively Spliced Events (MAASE) database system was formed to support splicing microarray functions. It consists of a manual/computational annotation tool for the proficient mining of crucial sequence and functional information of AS events and a user-friendly database of annotated events for the supply of data for microarray design and data analysis [54]. The AsMamDB database was generated to study AS genes in mammals. It provides information regarding AS patterns in genes, structure of genes, their location in the chromosome, and the gene products and tissues where expression takes place [87]. ASmodeler is a unique web-based tool for obtaining gene models including AS events from the genomic alignment of mRNA, EST and protein sequences [88]. The Alternative Splicing and Transcript Diversity database (ASTD) gives information about a huge collection of alternative transcripts combining transcription initiation, polyadenylation and splicing variant data and has now been intergrated into Ensembl genome browser [89].

ProSplicer is a database of acknowledged AS data obtained from the alignment of proteins, mRNA sequences and ESTs matched against human genomic sequences. Proteins, mRNA and ESTs provide important data that can divulge gene splice variants [90]. ASTRA is a database with a Java-based browser that can efficiently reorganize the order of displayed splicing patterns [91]. SpliceInfo is an information repository describing the incidence of the four main AS modes in the human genome which include exon skipping, 5’-AS, 3’-AS and intron retention. Data is obtained by comparing nucleotide and protein sequences for a given gene for any evidence of AS. Added features including tissue specificity, protein domain contained by exons, GC-ratio of exons, repeats present within the exons and gene ontology are annotated computationally for each exonic region containing AS [92]. In 2001, three independent databases GeneNest, SpliceNest and SYSTERS which are available online, were linked to each other and to other major databases. This collaboration has helped researchers in exploring the entire sequences of many proteins, mRNA, ESTs and genomic DNA [93]. Putative alternate splicing database (PALS db) contains information from 19,936 human UniGene clusters and 16,615 mouse UniGene clusters. It predicts AS sites by using the longest mRNA sequence in each UniGene cluster as the reference sequence and then aligning it with a linked sequence in UniGene and dbEST [94]. Human-transcriptome DataBase for Alternative Splicing (H-DBAS) is a unique database of AS human transcripts [95].

ECgene provides functional annotation for alternatively spliced genes. It includes the genome-based transcript modeling for alternative splicing (AS), domain analysis with Gene Ontology (GO) annotation and expression analysis based on the EST and Serial analysis of gene expression (SAGE) data. ECgene has AS modeling and EST clustering includes nine organisms for which sufficient EST data is available in the GenBank. ECgene has also launched several new applications to analyze differential expression for the human genome [96]. H-InvDB was started in 2004 and in their latest version, H-InvDB 8.0, mapping of 244 709 human complementary DNA was performed onto the hg19 reference genome and 43 829 gene loci, including nonprotein-coding ones, were identified [97]. Alternative splicing transcriptional landscape visualization tool (ASTALAVISTA) utilizes an intuitive and complete notation system to clearly recognize AS events. It can characterize AS from data for entire transcriptomes from reference annotations (GENCODE, REFSEQ, ENSEMBL) and also for genes selected by the user based on common functional/structural attributes of interest [98]. Alternative splicing prediction database (ASPicDB) provides the means for splice-site detection and full-length transcript modelling by a genome-wide application of the ASPic algorithm that uses the multiple alignments of gene-related transcripts (typically a Unigene cluster) to the genomic sequence. It also provides information on tissue-specific splicing patterns of normal and cancer cells by looking at data on available EST sequences and their library source annotation [99]. DBASS3 and DBASS5 offer comprehensive data of new exon boundaries induced by pathogenic mutations in human disease genes. They currently contain approximately 900 records of cryptic and de novo 3’- and 5’-splice sites that were produced by over a thousand different mutations in approximately 360 genes [100].

Alternative splicing electronic RT-PCR (ASePCR) is a web-based tool that emulates RT–PCR in various tissues. NCBI provides a reverse e-PCR option which generates transcript models and ASePCR can then estimate the amplicon size for a given primer pair based on these transcript models. The tissue specificity of each PCR band is inferred from the tissue data of EST sequences compatible with each transcript structure. The result generated in the output page is in the form of PCR bands in a gel electrophoresis in various tissues. Each band characterizes a putative isoform that may occur in a tissue-specific manner also providing the EST alignment and tissue information in the genome browser. Also, the AS patterns of orthologous genes in other species can be compared [101].

SNPSplicer helps in the quick analysis of experiments involving screening of SNPs located in sequences of splicing relevance. It proposes to use sets of matching DNA and complementary DNA (cDNA) as a screening tool to detect the potential splice effects of SNPs in RT-PCR experiments with tissue material from genotyped sources [102]. SplicingViewer is a very useful tool for the clear detection, annotation and visualization of splice junctions and alternative splicing events from RNA-Seq data [103]. SpliceGrapher is another tool that aids in discriminating between real and false splice sites and can improve the reliability of identification of AS for RNA-Seq data [104]. TopHat is another proficient algorithm programmed to align reads from an RNA-Seq experiment to a reference genome without depending on known splice sites [105]. Multivariate analysis of transcript splicing (MATS) is a Bayesian statistical framework used for RNA-Seq data analysis. It is used for flexible hypothesis testing of differential alternative splicing patterns. The MATS method is useful for almost any type of null hypotheses of interest, giving the flexibility to identify differential alternative splicing events that match a given user-defined pattern [106]. PASSion is a pattern growth algorithm-based pipeline useful in the detection of splice sites in paired-end RNA-Seq reads and can detect junctions that do not have known splicing motifs, which cannot be found by the other tools [107]. SpliceTrap is a method to quantify inclusion levels of exon utilizing paired-end RNA-seq data. Contrasting other tools that focus on full-length transcript isoforms, SpliceTrap looks at the expression-level estimation of each exon as an independent Bayesian inference problem. Also, it can identify key classes of alternative splicing events under a single cellular condition, without the need for a background set of reads to estimate relative splicing changes [108].

AltAnalyze and the Cytoscape plugin DomainGraph programs offer an intuitive and comprehensive solution for the analysis and visualization of AS data from Affymetrix exon and gene arrays at the level of proteins, domains, microRNA binding sites, molecular interactions and pathways [109]. Junction and Exon Toolkits for Transcriptome Analysis (JETTA) is an integrated software tool for calculating gene expression indices and also aids in the identification and visualization of AS events [110]. Cufflinks is another useful bioinformatic tool that does not use existing gene annotations during the assembly of transcripts but assembles a minimum set of transcripts that describe the reads in the dataset in the best way [111]. Alternative Splice Site Predictor (ASSP) is a web tool used for predicting alternative splice sites [112]. GeneSplicer is a flexible bioinformatic system employed in identifying splice sites in the genomic DNA of various eukaryotes [113]. MapSplice is a second generation splice detection algorithm targeting high sensitivity and specificity for detecting splices and also CPU and memory efficiency. It can be applied to both short (<75 bp) and long reads (>75 bp) and does not depend on splice site features or intron length, thus it can identify novel canonical as well as non-canonical splices [114]. Mixture-of-isoforms (MISO) model is a statistical model used in the estimation of expression of alternatively spliced exons and isoforms and also evaluates confidence in these estimates. MISO is very valuable to infer isoform regulation from high-throughput sequencing of cDNA fragments (RNA-Seq) [115]. The PASA software [116] gathers and clusters spliced transcript alignments, offering transcript-based gene structures that are used to automatically improve existing gene annotations by adding untranslated regions (UTRs), adjusting intron and exon boundaries and adding new models representing alternative splicing from its many other functions [117]. DEXSeq is a statistical method used to analyze RNA-Seq data by testing for differential exon usage with high sensitivity [118]. HMMSplicer performs particularly well in compact genomes and on genes with low expression levels, alternative splice isoforms, or non-canonical splice junctions [119].

## 5 CONCLUSION

AS is a vital mechanism regulating gene expression in multicellular organisms. Transcriptional regulation of the promoter mainly results in variations in the amount of RNA generated, producing N-terminal protein variants by different transcriptional start sites. However, regulation through AS is much more flexible with alterations in the protein sequence potentially affecting almost all areas of protein function including binding properties, enzyme activity, intracellular localization, protein stability, phosphorylation and glycosylation patterns. AS-site regulation is obtained by combining many weak interactions between regulatory proteins and signals on the pre-mRNA [7].

Changes in the selection of splice sites may arise not only due to disease but also to the natural adaptation of a cell. Many stimuli including growth factors, cytokines, hormones and cell depolarization cause altered splice site selection [120]. Sequence analyses predict that only around 10% of AS events are likely to delete, insert or modify functional domains in proteins and these events give a fairly straightforward approach to design suitable experiments for addressing the functional consequences of AS [10, 121]. As for the remaining 90% of AS events found in coding regions, they do not overlap with boundaries of functional protein domains or regions which are vital for overall protein folding [10]. Most AS events influence coiled or “loop” regions of secondary protein structures and the greater part of these coding sequences are situated on protein surfaces [122]. These regions help perform protein-protein interactions or interactions with other types of ligands [10].

After sequencing of the genome by the human genome project, it is now possible to examine the evolutionary effects and limitations of AS. AS provides a vital strategy in understanding pathways that are applicable in the evolution of gene structures and in uncovering novel protein-coding sequences. Mammalian genomes give a broad indication of RNA selection pressure due to the restriction of AS regulation, thus highlighting the functional significance of AS patterns [123].
